# Does competitive swimming affect lung growth?

**DOI:** 10.14814/phy2.13816

**Published:** 2018-08-06

**Authors:** Joshua M. Bovard, Joseph F. Welch, Kristin M. Houghton, Donald C. McKenzie, James E. Potts, Andrew William Sheel

**Affiliations:** ^1^ School of Kinesiology University of British Columbia Vancouver Canada; ^2^ Division of Pediatrics Faculty of Medicine University of British Columbia Vancouver Canada; ^3^ Division of Sports Medicine Faculty of Medicine University of British Columbia Vancouver Canada

**Keywords:** swim, exercise, lung growth, puberty, ventilatory constraints

## Abstract

Whether the large lungs of swimmers result from intensive training or genetic endowment has been widely debated. Given that peak lung growth velocities occur during puberty, this study examined if competitive swimming during puberty affected lung growth. Eleven‐ to fourteen‐year‐old healthy female competitive swimmers and controls were assessed before (PRE) and after (POST) one swimming season (7.4 ± 0.5 months). Pulmonary function testing included lung volumes, spirometry, diffusion capacity (D_L__,_ _CO_), and maximal inspiratory (PI_MAX_) and expiratory (PE_MAX_) pressures. Ventilatory constraints, including end‐expiratory lung volume, expiratory flow limitation, and utilization of ventilatory capacity, were assessed during an incremental cycling test. Swimmers (*n* = 11) and controls (*n* = 10) were of similar age, size, and sexual maturity (*P* > 0.05). However, swimmers compared to controls had a greater total lung capacity (PRE 4.73 ± 0.73 vs. 3.93 ± 0.46, POST 5.08 ± 0.68 vs. 4.19 ± 0.64 L; *P* < 0.01), peak expiratory flow (PRE 6.48 ± 0.92 vs. 5.70 ± 0.86, POST 6.97 ± 0.84 vs. 6.00 ± 0.77 L·s^−1^; *P* = 0.03), and PE_MAX_ (*P* < 0.001). Although D_L__,_ _CO_ was greater in swimmers (*P* = 0.01), differences were attenuated when expressed relative to alveolar volume (PRE 5.14 ± 0.60 vs. 5.44 ± 0.44, POST 4.91 ± 0.56 vs. 5.16 ± 0.38 mL min^−1^ mmHg^−1^ L^−1^; *P* = 0.20). The groups achieved a similar maximal oxygen uptake (*P* = 0.32), and ventilatory constraints experienced were not different (*P* > 0.05). Changes over time were not different between groups (*P* > 0.05). At the initial measurement, pubertal female swimmers had greater lung size, expiratory flows, and indices of respiratory muscle strength, but similar ventilatory constraints while cycling. One competitive swimming season did not further accentuate this enhanced lung size and function or alter ventilatory mechanics, suggesting that competitive swimming during puberty did not affect lung growth.

## Introduction

The pulmonary size and function of competitive swimmers is characterized by greater lung capacities (Astrand et al. 1963; Andrew et al. 1972; Baxter‐Jones and Helms 1996), expiratory flows (Andrew et al. 1972; Armour et al. 1993; Courteix et al. 1997), and diffusion capacities (Yost et al. 1981; Armour et al. 1993) compared to healthy controls and predicted values. However, it has been widely debated whether this enhanced pulmonary profile is an adaptation to swim training (Zauner and Benson 1981; Bloomfield et al. 1990; Courteix et al. 1997), the result of self‐selection into swimming based on favorable genetic endowments (Baxter‐Jones and Helms 1996), or both (Andrew et al. 1972; Zinman and Gaultier 1986, 1987).

If the enhanced pulmonary profile of swimmers is an adaptation to training, effects are likely to be most evident during critical periods of maximal lung development that occur before 7 years old (Mansell et al. 1977) and during puberty (Sherrill et al. 1989). Swimmers younger than 7 years old have not been examined. While greater lung capacities were observed in large cohorts of swimmers as young as 7–9 years old (Andrew et al. 1972; Zinman and Gaultier 1986; Baxter‐Jones and Helms 1996), other studies reported no differences (Engstrom et al. 1971; Vaccaro and Clarke 1978; Zauner and Benson 1981). The few studies focusing on changes during puberty have also presented opposing findings. Some found that forced vital capacity (FVC) (Engstrom et al. 1971; Bloomfield et al. 1990) and total lung capacity (TLC) (Zinman and Gaultier 1987) increased more than can be explained by somatic growth alone, whereas others have not (Engstrom et al. 1971; Baxter‐Jones and Helms 1996).

Longitudinal analyses of competitive swimmers during growth have also reported conflicting results. First, no differences in FVC were found before or after 7 months of intensive swim training in 9‐ to 11–year‐old children compared to age‐matched controls (Vaccaro and Clarke 1978). Second, despite similar lung capacities initially, greater lung capacities were measured in swimmers compared to either a control group or predicted values after 1–5 years of training (Zauner and Benson 1981; Bloomfield et al. 1990; Courteix et al. 1997). Third, swimmers initially had larger lungs that did not increase further over 1–5 years of training compared to other athletes, nonathletes, or predicted values (Engstrom et al. 1971; Andrew et al. 1972; Baxter‐Jones and Helms 1996). Lastly, trained swimmers had larger lungs initially which increased further after 1 year of training (Zinman and Gaultier 1987). Thus, while there are many published reports on competitive swimming and lung development, considerable between‐study heterogeneities in chronological age, competitive status, study length, control groups, and experimental design and analysis have led to varying conclusions. Therefore, it remains equivocal if swim training enhances lung growth or if the larger lungs of swimmers reflect an inherent predisposition leading to self‐selection into the sport.

Furthermore, whether competitive swimming affects ventilatory constraints during exercise has not been studied and warrants investigation. Ventilatory constraints reflect mechanisms that oppose the requirement for ventilation and induce a reduction in, but do not necessarily limit, the ventilatory response (Whipp and Pardy 2011). Since ventilatory capacity (${\dot{V}}_{\mathrm{ECAP}}$) is primarily determined by anatomical features, including lung size (Green et al. 1974), the larger lung volumes and expiratory flows of swimmers may be advantageous during exercise if it leads to a larger ${\dot{V}}_{\mathrm{ECAP}}$. Swimmers may then be less susceptible to ventilatory constraints during exercise, or achieve increased metabolic and ventilatory demands within similar ventilatory constraints. Adolescence is a critical period during which physiological changes can significantly influence health throughout the lifespan (Harms et al. 2011), such as years of competitive swim training leading to improvements in lung function that persist into adulthood (Eriksson et al. 1978). Thus, studying changes in lung development and susceptibility to ventilatory constraints will provide a greater understanding of the potential benefits of competitive swimming during youth.

The aim of this study was to determine if one season of competitive swimming affected lung growth in pubertal females. Given the age range at which peak growth velocities occur for FVC (Sherrill et al. 1989) and lung and chest wall dimensions (Simon et al. 1972), we compared 11‐ to 14‐year‐old female competitive swimmers and healthy female controls of similar age, size, and maturation. Our specific purposes were threefold. First, to compare initial differences in lung size and function. Second, to determine if one season of competitive swimming affected the development of lung size and function. Lastly, to characterize ventilatory mechanics during cycling exercise.

## Methods

### Subjects

Healthy female swimmers (SWIM) and controls (CON) underwent pulmonary function and incremental exercise testing at BC Children's Hospital before (PRE) and after (POST) one swimming season. Swimmers were recruited from Vancouver swim clubs and competed regionally, provincially, or nationally. Controls participated primarily in gymnastics, dance, and team sports, but did not perform any sport‐specific endurance training. Subjects had no reactive airway disease (e.g., asthma), previous use of an inhaler, or previous exposure to high altitude for a period greater than 6 weeks. All subjects provided written informed assent and were accompanied by a guardian who provided necessary medical history and written informed consent. All procedures received institutional ethical approval, which conformed to the *Declaration of Helsinki*.

### Experimental overview

Subjects self‐assessed their sexual maturity using a validated form (Morris and Udry 1980) corresponding to Tanner's five pubertal stages. Certified pediatric Respiratory Therapists measured each subject's height (seca 217, seca GmbH & Co. KG., Hamburg, Germany), weight (Scale‐Tronix, White Plains, NY), and noninvasive hemoglobin (via CO‐oximetry; Pronto‐7, Masimo Corp., Irvine, CA), and performed the pulmonary function test consisting of spirometry, lung volumes, and diffusion capacity (MasterScreen™ PFT system, Jaeger, CareFusion Corp., San Diego, CA). Maximal inspiratory (PI_MAX_) and expiratory (PE_MAX_) pressure maneuvers (Mouth Pressure Meter, Micro Direct, Inc., Lewiston, ME) were then performed. Resting baseline data were collected while seated on a cycle ergometer (Excalibur Sport, Lode BV, Groningen, Netherlands), which was followed by the incremental cycling test. After the second visit, physical activity levels were self‐assessed with a modified version of a validated physical activity questionnaire (Kowalski et al. 2004). Training details were collected from each swimmer's coach.

### Pulmonary function

Using the single‐breath technique, hemoglobin‐corrected (MacIntyre et al. 2005) diffusion capacity for carbon monoxide (D_L,CO_) and alveolar volume (*V*_A_) were measured by carbon monoxide diffusion; helium dilution was used to determine functional residual capacity (FRC), TLC, vital capacity, and residual volume (RV). Spirometry was used to determine FVC, forced expiratory volume in one second (FEV_1_), FEV_1_/FVC, peak expiratory flow (PEF), and forced expiratory flows (FEF). All measurements were performed in the seated position with nose clips according to established guidelines (American Thoracic Society/European Respiratory Society, 2002; MacIntyre et al. 2005; Miller et al. 2005; Wanger et al. 2005) and compared to predicted values (Hibbert et al. 1989; Domenech‐Clar et al. 2003; Kim et al. 2012).

### Incremental maximal exercise test

Subjects sat on a cycle ergometer and breathed quietly through a two‐way non‐rebreathing valve (#2700B, Hans Rudolph, Kansas City, MO) connected to a customized metabolic cart. The cart consisted of independently calibrated pneumotachographs (#3813, Hans Rudolph, Kansas City, MO) and a mixing chamber for expired gases connected to O_2_ and CO_2_ analyzers (VacuMed #17625 and 17630, VacuMed, Ventura, CA). Heart rate (HR) was measured using a noncoded chest transmitter (T34, Polar Electro, Kempele, Finland).

After 5 min of seated rest, subjects performed inspiratory capacity (IC) maneuvers and multiple FVC and graded FVC maneuvers. The FVC maneuvers were performed according to guidelines (Miller et al. 2005), whereas the graded FVC maneuvers were performed with extensive coaching to ensure the subjects inspired maximally to TLC and expired to RV at various degrees of submaximal effort (Dominelli and Sheel 2012). Both maneuvers were repeated following the exercise test. Individual maximum expiratory flow volume (MEFV) curves were created by superimposing all FVC and graded FVC maneuvers and determining the highest flow for each 10 mL increment of the FVC. Numerical descriptions of the MEFV curve, including the *β*‐angle (describing the curvature), flow ratio (describing the curvature at low lung volumes), and slope ratio (describing the emptying of the lungs), were calculated using methods previously described (Dominelli et al. 2015).

Subjects warmed‐up for 3 min at 20 W, then started the test at 40 W and increased stepwise by 20 W every 2 min. Subjects were instructed to maintain a pedaling frequency of 60 rpm throughout the test, which was terminated when the subject could no longer maintain 50 rpm for at least 5 sec despite strong verbal encouragement. During each stage, two IC maneuvers were performed and a rating of perceived exertion was provided using the validated, 10‐point OMNI scale for children and adolescents that incorporates both pictorial and verbal descriptors of exertion (Robertson et al. 2000).

### Metabolic data

Metabolic data, including tidal volume (*V*_T_), breathing frequency (*f*_B_), minute ventilation (${\dot{V}}_{E}$), oxygen uptake ($\dot{V}$O_2_), and carbon dioxide production ($\dot{V}$CO_2_), were recorded continuously using an analog‐to‐digital data acquisition system (PowerLab/16SP model ML 795, ADInstruments, Colorado Springs, CO) and presented as the 20–30 sec average before the IC maneuver. Maximal exercise data preceded the final IC maneuver and maximal oxygen uptake ($\dot{V}$O_2MAX_) was compared to reference values (Cooper et al. 1984).

### Ventilatory mechanics

Ventilatory mechanics were assessed quantitatively using operational lung volumes, severity and frequency of expiratory flow limitation (EFL), and utilization of ${\dot{V}}_{\mathrm{ECAP}}$ (${\dot{V}}_{E}$/${\dot{V}}_{\mathrm{ECAP}}$) (Dominelli and Sheel 2012). End‐expiratory lung volume (EELV) was determined from EELV = FVC – IC, end‐inspiratory lung volume (EILV) from EILV = EELV + *V*_T_, and inspiratory reserve volume (IRV) from IRV = FVC–EILV. The severity of EFL was determined from the amount of overlap between the tidal flow–volume loop (FVL) (see below) and MEFV curve. For each 10 mL increment of the *V*_T_, increments whereby the FVL's expiratory flow was greater than the MEFV curve were considered flow limited. When ≥5% of the increments were flow limited, EFL was deemed present (Chapman et al. 1998). Lastly, ${\dot{V}}_{\mathrm{ECAP}}$ was calculated for each stage as the theoretical maximum ${\dot{V}}_{E}$ based on the subject breathing at the maximum expiratory flow for the entire tidal breath, as described elsewhere (Johnson et al. 1995).

Ventilatory mechanics were also assessed qualitatively by superimposing the FVL on the MEFV curve. The FVL was generated for each stage by averaging flows over 10 mL increments of the *V*_T_ from tidal breaths in the same 20–30 sec period preceding the IC maneuver as was used for the metabolic data. Thus, FVL were composed of a minimum of 5 tidal breaths during resting baseline to a maximum of 30 tidal breaths during maximal exercise. The FVL were then superimposed onto the MEFV curve by aligning the *V*_T_ with the EELV. Composite MEFV curves and FVL were created for each group to compare group responses.

### Statistical analysis

Two‐way mixed‐factorial analysis of variance (ANOVA) tests compared groups and time points for descriptive characteristics, pulmonary function, MEFV curve quantities, and $\dot{V}$O_2MAX_. Three‐way mixed‐factorial ANOVA compared exercise responses between groups, time points, and relative work rates. Levene's test assessed homogeneity of variances between the groups. Mauchly's sphericity test determined if the variances of the differences between levels of the within‐subject factors for both groups were equal. When a significant difference rejected the assumption of sphericity, the Greenhouse–Geisser correction was interpreted. Statistically significant *F*‐ratios were further analyzed for magnitude and direction using independent and paired *t*‐tests for between‐ and within‐subject data, respectively. Main effects were not interpreted if significant interactions were found.

Sexual maturity rating and frequency of EFL were assessed with generalized estimating equations (Ballinger 2004). Independent *t*‐tests compared the time between visits and self‐reported physical activity levels. Associations between swimming history and pulmonary function, as well as swim training volume and changes in pulmonary function, were quantified using Pearson's correlation coefficient. For all statistical tests, normality was assessed qualitatively by visually inspecting descriptive statistics, histograms, and quantile–quantile (Q‐Q) plots and quantitatively using a suitable Shapiro–Wilk test for small samples. A level of significance of *P* < 0.05 was used. Statistical tests were performed with SPSS (Version 20, IBM Corporation, Armonk, NY).

## Results

### Subject characteristics

Anthropometric, lung function, and MEFV curve data for swimmers (*n* = 11) and controls (*n* = 10) are presented in Table 1. The duration between visits was 7.3 ± 0.5 for SWIM and 7.6 ± 0.4 months for CON (*P* = 0.16). Groups were of similar age, height, weight, and sexual maturity rating (*P* > 0.05), all of which significantly increased from PRE to POST (*P* < 0.001). Moreover, 100% of SWIM and 90% of CON reported their sexual maturity rating to be pubertal (i.e., in Tanner stages 2–4) at PRE or POST. Hemoglobin values were within normal ranges and equal between groups (*P* = 0.89). Self‐reported daily moderate‐vigorous physical activity (121 ± 25 vs. 110 ± 55 min, *P* = 0.76) and physical activity levels based on the modified physical activity questionnaire score (3.1 ± 0.4 vs. 3.1 ± 0.5, *P* = 0.58) were not different between groups. For swimmers, the onset of training varied from 6.0 to 10.1 years old and experience ranged from 1.1 to 6.3 years. They swam 5–7 times per week for an average of 9.1 ± 3.6 h and 19 ± 8 km. To note, one swimmer trained predominantly for water polo during the study period, but was still included because training involved 12 h per week of water polo, weekly speed swimming sessions, and swim meets during the summer season.

**Table 1:** Anthropometric, lung function, and maximum expiratory flow–volume curve data

	SWIM (*n* = 11)		CON (*n* = 10)	
	PRE	POST	PRE	POST
Age (years)	12.4 ± 0.8	13.0 ± 0.8^a^	13.2 ± 1.3	13.8 ± 1.3
Height (cm)	161.3 ± 7.9	163.4 ± 6.9^a^	158.3 ± 7.4	160.7 ± 7.0
Weight (kg)	52.4 ± 10.8	55.8 ± 9.8^a^	46.3 ± 5.4	49.4 ± 5.6
Sexual maturity rating	4 (2–4)	4 (3–5)^a^	3 (1–5)	4 (3–5)
Hemoglobin (g dL^−1^)	13.3 ± 1.5	13.8 ± 1.0	13.5 ± 0.6	13.7 ± 1.3
TLC (L)	4.73 ± 0.73^b^	5.08 ± 0.68^a^	3.93 ± 0.46	4.19 ± 0.64
TLC (% predicted)	110 ± 7^b^	112 ± 8	94 ± 7	94 ± 7
FRC (L)	2.18 ± 0.43	2.40 ± 0.39^c^	2.19 ± 0.28	2.21 ± 0.40
FRC (%‐predicted)	102 ± 14	106 ± 14	103 ± 4	96 ± 7^c^
RV (L)	0.99 ± 0.16	1.04 ± 0.19^a^	0.96 ± 0.20	1.01 ± 0.25
RV (%‐predicted)	96 ± 13	95 ± 16	91 ± 14	90 ± 16
FRC/TLC (%)	46 ± 5^d^	47 ± 6	56 ± 4	53 ± 5
RV/TLC (%)	21 ± 3	21 ± 3	24 ± 5	24 ± 5
FVC (L)	3.92 ± 0.71^b^	4.15 ± 0.61^a^	3.13 ± 0.50	3.28 ± 0.54
FVC (%‐predicted)	123 ± 11^b^	125 ± 10	102 ± 11	101 ± 11
FEV_1_/FVC (%)	85 ± 2	85 ± 3	84 ± 7	84 ± 7
PEF (L s^−1^)	6.48 ± 0.92^b^	6.97 ± 0.84^a^	5.70 ± 0.86	6.00 ± 0.77
PEF (%‐predicted)	97 ± 9^b^	101 ± 8	86 ± 10	87 ± 10
FEF_25–75%_ (L sec^−1^)	3.56 ± 0.73^b^	3.76 ± 0.84^a^	2.74 ± 0.81	2.85 ± 0.83
FEF_25–75%_ (%‐predicted)	100 ± 15^b^	101 ± 15	79 ± 22	78 ± 22
D_L,CO_ (mL min^−1^ mmHg^−1^)	23.43 ± 2.58^b^	24.09 ± 1.83	20.73 ± 1.88	21.00 ± 3.18
D_L,CO_ (%‐predicted)	122 ± 12^b^	121 ± 13	110 ± 8	107 ± 9
D_L,CO_/*V*_A_ (mL min^−1^ mmHg^−1^ L^−1^)	5.14 ± 0.60	4.91 ± 0.56^a^	5.44 ± 0.44	5.16 ± 0.38
D_L,CO_/*V*_A_ (%‐predicted)	101 ± 10	97 ± 9	106 ± 8	101 ± 8
PI_MAX_ (cm H_2_O)	87 ± 26	103 ± 22^a^	71 ± 24	79 ± 26
PI_MAX_ (%‐predicted)	96 ± 26	109 ± 19^a^	82 ± 30	87 ± 30
PE_MAX_ (cm H_2_O)	112 ± 17^b^	127 ± 17^a^	85 ± 16	98 ± 18
PE_MAX_ (%‐predicted)	104 ± 13^b^	114 ± 13^a^	77 ± 19	84 ± 19
Flow ratio (%)	−3 ± 19	−5 ± 17	−5 ± 8	0 ± 10
*β*‐angle (°)	195 ± 9	194 ± 14	194 ± 15	191 ± 12
Slope ratio (au)	0.83 ± 0.23	0.83 ± 0.21	0.89 ± 0.23	0.96 ± 0.23

Values presented as mean ± SD except sexual maturity rating (presented as median (range)). Significant interactions were found for FRC, FRC (%‐predicted), and FRC/TLC.

SWIM, swimmers; CON, controls; TLC, total lung capacity; FRC, functional residual capacity; RV, residual volume; FVC, forced vital capacity; PEF, peak expiratory flow; D_L,CO_, diffusion capacity for carbon monoxide; *V*_A_, alveolar volume; PI_MAX_, maximal inspiratory static mouth pressure; PE_MAX_, maximal expiratory static mouth pressure.

a *P* < 0.05, main effect PRE versus POST.

b *P* < 0.05, main effect SWIM versus CON.

c *P* < 0.05, within group PRE versus POST.

d *P* < 0.05, within time point SWIM versus CON.

### Pulmonary function

**Figure 1:**
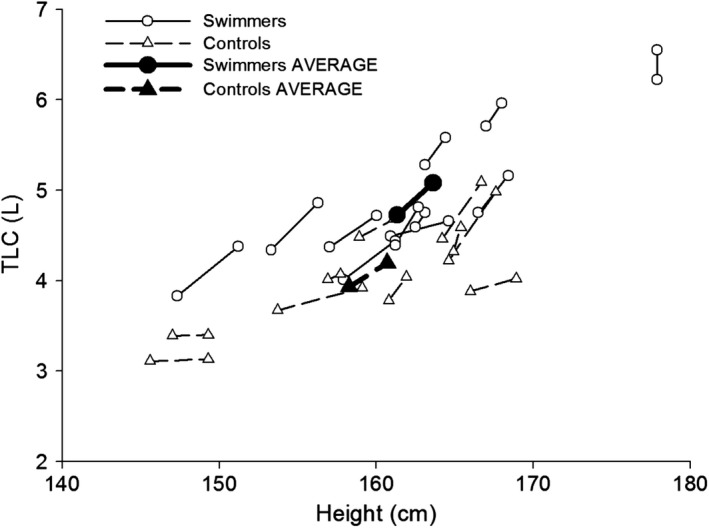
Total lung capacity (TLC) for individual subjects in relation to their height. Individual data are presented with an open symbol, whereas group average have a closed symbol. Data are connected by a solid line for swimmers and a hashed line for controls.

**Figure 2:**
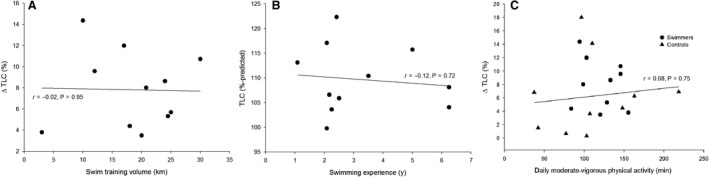
Changes in total lung capacity (TLC). (A) Relative change in TLC from PRE to POST compared to the average weekly swim training volume (km) for each swimmer (the subject who swam 3 km per week trained for water polo 12 h per week, as noted in text). (B) Percent‐predicted TLC at the initial measurement compared to the number of years of swimming experience for each swimmer. (C) Relative change in TLC from PRE to POST compared to the average daily moderate‐vigorous physical activity in all subjects.

**Figure 3:**
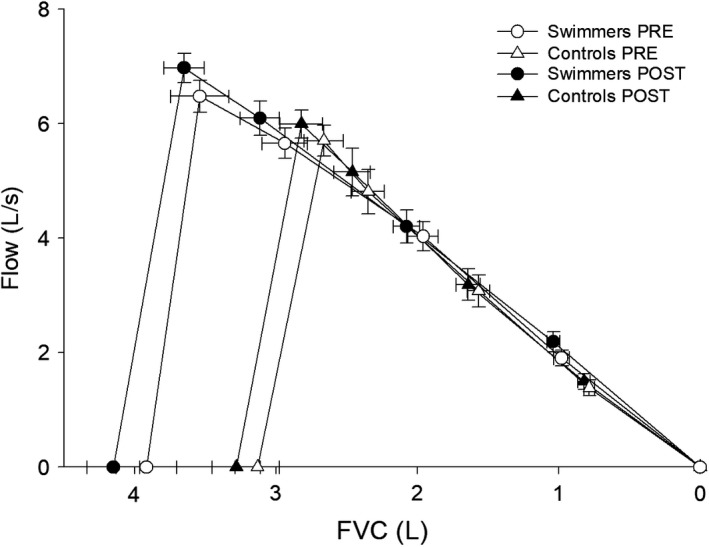
Average maximum expiratory flow–volume curve from the pulmonary function test. Data points are presented as mean ± SE. From left to right, data points represent total lung capacity, peak expiratory flow (PEF), forced expiratory flow when 25% (FEF_25%_), 50% (FEF_50%_), and 75% (FEF_75%_) of the forced vital capacity (FVC) has been expired, and residual volume (RV).

Individually, swimmers had a greater TLC for nearly all heights (Fig. 1). As a group, swimmers had a greater TLC (*P* < 0.01) that was already ~800 mL and 20% greater than controls at the initial measurement. However, there was no significant interaction between group and time point (*P* = 0.29) and no association was found between training volume and relative change in TLC (*r* = −0.02, *P* = 0.95) (Fig. 2A). Swimmers consistently exceeded their predicted values for many measures at PRE, including TLC (range 100–122%), FVC (106–143%), and D_L,CO_ (100–142%), but there was no association between %‐predicted TLC and swimming experience (*r* = −0.12, *P* = 0.72) (Fig. 2B) or starting age of swimming (*r* = 0.20, *P* = 0.56). Swimmers also had greater FVC (*P* < 0.01), PEF (*P* < 0.01), and forced expiratory flows (Table 1). However, the average MEFV curve in Figure 3 shows that both groups produced similar flows for a given absolute FVC. Although D_L,CO_ was greater in SWIM (*P* = 0.01), there was no difference when expressed relative to *V*_A_ (*P* = 0.20). Both PI_MAX_ (*P* = 0.06) and PE_MAX_ (*P* < 0.001) were greater in SWIM. Changes from PRE to POST were similar between groups (interactions *P* > 0.05) for all pulmonary function measures except those including FRC (*P* < 0.04). When the swimmers and controls were combined into one group, no association was found between daily moderate‐vigorous physical activity levels and relative change in TLC (*r* = 0.08, *P* = 0.75) (Fig. 2C).

### Metabolic data

**Figure 4:**
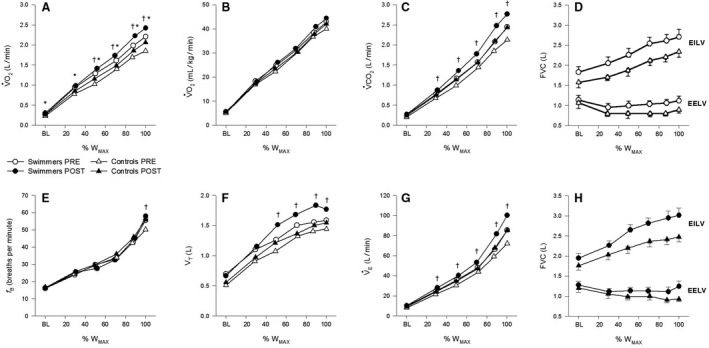
Mean metabolic and ventilatory responses during exercise. (A) Oxygen uptake ($\dot{V}O_{2}$), (B) $\dot{V}O_{2}$ relative to body mass, (C) carbon dioxide production ($\dot{V}{\mathrm{CO}}_{2}$), (D) end‐inspiratory (EILV) and end‐expiratory (EELV) lung volumes at PRE, (E) breathing frequency (*f*_B_), (F) tidal volume (*V*_T_), (G) minute ventilation (${\dot{V}}_{E}$), and (H) EILV and EELV at POST. Data points are presented as mean ± SE. All exercise stages were significantly increased from baseline except for EELV, which was significantly decreased from baseline for all stages except maximal exercise. % WMAX, relative work rate; BL, baseline. †*p*<0.05, PRE vs. POST. *p<0.05, SWIM vs. CON.

Metabolic and ventilatory variables during maximal exercise are presented in Table 2, and the exercise responses as relative work rate increased are displayed for selected variables in Figure 4. To note, exercise responses were compared against both relative and absolute work rates, and produced the same results. However, the large variation in subject size (range 36–72 kg) and peak work rate (range 120–220 W) meant that the highest equivalent submaximal work rate was only 100 W. Given that comparisons against absolute work rates did not include submaximal work rates greater than 100 W, whereas relative work rates spanned all submaximal exercises intensities, exercise data are presented against relative work rate.

**Table 2:** Metabolic and ventilatory responses at maximal exercise

	SWIM (*n* = 11)		CON (*n* = 10)	
	PRE	POST	PRE	POST
Duration (min)	14.3 ± 3.0	16.5 ± 1.5	12.8 ± 2.7	14.6 ± 2.7
Work rate (W)	167 ± 29	191 ± 16	154 ± 25	170 ± 27
Work rate (W kg^−1^)	3.3 ± 0.5	3.5 ± 0.6	3.3 ± 0.4	3.5 ± 0.5
HR (beats min^−1^)	192 ± 10	195 ± 8	196 ± 7	198 ± 8
RPE (OMNI scale)	9.3 ± 1.8	9.5 ± 1.0	9.3 ± 1.0	9.4 ± 0.9
*V*_T_ (L)	1.59 ± 0.33	1.7 ± 0.26	1.44 ± 0.25	1.55 ± 0.26
*f*_B_ (breaths min^−1^)	56 ± 16	58 ± 13	50 ± 8	56 ± 7
${\dot{V}}_{E}$ (L min^−1^)	86 ± 21	100 ± 18	72 ± 16	85 ± 13
$\dot{V}$O_2_ (L min^−1^)	2.20 ± 0.35	2.42 ± 0.23	1.85 ± 0.25	2.07 ± 0.27
$\dot{V}$O_2_ (mL kg^−1^ min^−1^)	42.9 ± 6.8	44.4 ± 8.1	40.1 ± 4.2	42.1 ± 5.2
$\dot{V}$O_2_ (%‐predicted)	125 ± 18	131 ± 21	115 ± 12	122 ± 14
$\dot{V}$CO_2_ (L min^−1^)	2.45 ± 0.42	2.77 ± 0.25	2.13 ± 0.38	2.43 ± 0.33
RER	1.11 ± 0.05	1.15 ± 0.05	1.15 ± 0.09	1.18 ± 0.06
${\dot{V}}_{E}$/$\dot{V}$O_2_	39 ± 5	42 ± 6	39 ± 6	42 ± 5
${\dot{V}}_{E}$/$\dot{V}$CO_2_	35 ± 4	36 ± 4	34 ± 4	35 ± 4
EILV/FVC (%)	80 ± 7	80 ± 7	84 ± 3	83 ± 6
EELV/FVC (%)	33 ± 5	33 ± 7	32 ± 7	31 ± 5
EFL (% *V*_T_)	19 ± 24	28 ± 28	13 ± 25	28 ± 21
${\dot{V}}_{\mathrm{ECAP}}$(L min^−1^)	123 ± 39	133 ± 38	110 ± 33	105 ± 21
${\dot{V}}_{E}$/${\dot{V}}_{\mathrm{ECAP}}$ (%)	73 ± 19	80 ± 21	69 ± 23	82 ± 13

Values presented as mean ± SD.

HR, heart rate; RPE, rating of perceived exertion; *V*_T_, tidal volume; *f*_B_, breathing frequency; ${\dot{V}}_{E}$, minute ventilation; $\dot{V}$O_2_, oxygen uptake; $\dot{V}$CO_2_, carbon dioxide production; RER, respiratory exchange ratio; EILV, end‐inspiratory lung volume; FVC, forced vital capacity; EELV, end‐expiratory lung volume; EFL, expiratory flow limitation; ${\dot{V}}_{\mathrm{ECAP}}$, ventilatory capacity.

At a given relative intensity, absolute work rate tended to be higher in swimmers (*P* = 0.10); however, when work rate was expressed relative to body mass, there was no difference between groups (*P* = 0.83). Thus, the slightly larger size and therefore absolute work rate of the swimmers may have led to the greater $\dot{V}$CO_2_ (*P* = 0.02), stimulating an increased ${\dot{V}}_{E}$ (*P* = 0.02). The latter was achieved by utilizing an identical *f*_B_ (*P* = 0.99) but larger *V*_T_ (*P* = 0.02). Differences in *V*_T_ were abolished when expressed relative to FVC (*P* = 0.32). Although there was a significant interaction between group and relative work rate for $\dot{V}$O_2_ (*P* = 0.02), whereby $\dot{V}$O_2_ was greater in SWIM at each stage (*P* < 0.05), these differences were negated when expressed relative to body mass (*P* = 0.26). Therefore, absolute $\dot{V}$O_2MAX_ was greater in swimmers initially (*P* < 0.01) and at the follow‐up measurement (*P* < 0.001), but there were no differences in relative $\dot{V}$O_2MAX_ between groups (*P* = 0.32) or time points (*P* = 0.11). There were no significant three‐ or two‐way interactions involving both group and time point.

### Ventilatory mechanics

**Figure 5:**
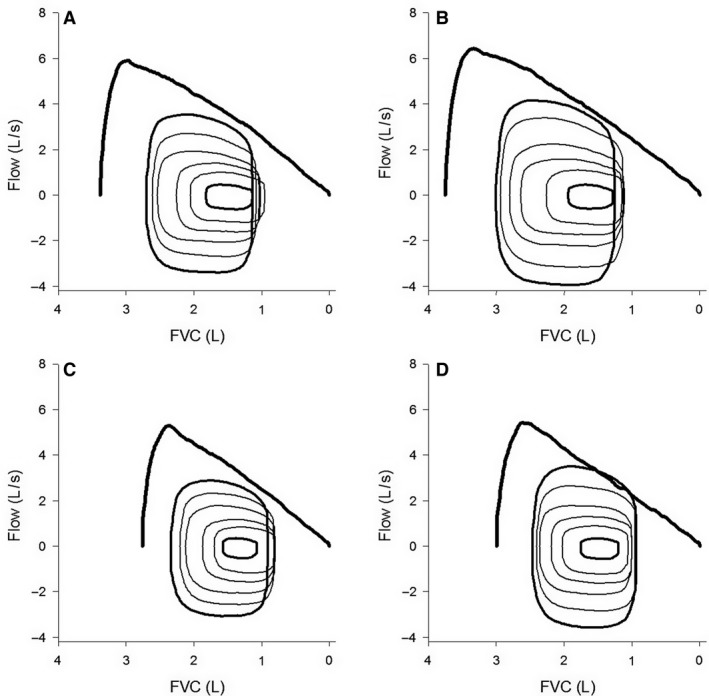
Composite maximum expiratory flow–volume curves and tidal flow–volume loops. (A) swimmers PRE, (B) swimmers POST, (C) controls PRE, and (D) controls POST. FVC, forced vital capacity.

Operational lung volumes are shown in Figures 4D and H. Two‐way interactions between group and relative work rate approached significance for EILV (*P* = 0.10), EELV/FVC (*P* = 0.07), and IRV (*P* = 0.10). A main effect of swimming was found for IRV (*P* < 0.01), whereas differences in EILV (*P* = 0.05), EILV/FVC (*P* = 0.08), and IRV/FVC (*P* = 0.08) also approached significance. Swimmers utilized a similar EELV (*P* = 0.18). Although there was a significant interaction between group and time point for IC (*P* = 0.02), IC was greater in SWIM compared to CON at PRE and POST (both *P* < 0.001). No other significant three‐ or two‐way interactions were found involving group and time point. The frequency of EFL was very low during submaximal exercise; no subject experienced EFL at <70% peak work rate, only one subject experienced EFL at <85% peak work rate, and only 1/11 and 4/11 swimmers and 2/10 and 3/10 controls experienced EFL at ~90% peak work rate at PRE and POST, respectively. At maximal exercise, 5/11 and 6/11 swimmers and 3/10 and 8/10 controls experienced EFL at PRE and POST, respectively. Thus, the frequency of EFL at maximal exercise was similar between groups (*P* = 0.72), but increased from PRE to POST (*P* = 0.03). The severity of EFL was 0% at baseline and 30%, 50%, and 70% of peak work rate. At 90% peak work rate, the severity was 5 ± 16 and 13 ± 23% for swimmers and 9 ± 19 and 11 ± 17% for controls at PRE and POST, respectively. Thus, severity of EFL was not different between groups throughout exercise (*P* = 0.95), nor was ${\dot{V}}_{\mathrm{ECAP}}$ (*P* = 0.23) and ${\dot{V}}_{E}$/${\dot{V}}_{\mathrm{ECAP}}$ (*P* = 0.96). Composite MEFV curves and FVL are shown in Figure 5.

## Discussion

### Main findings

The aim of this study was to longitudinally assess lung growth in pubescent, female swimmers compared to healthy controls of similar age, size, and maturation. The main findings were threefold. First, prior to a season of swim training, swimmers already had an enhanced pulmonary profile relative to both predictive values and controls. The greater lung size (i.e., TLC) and function (i.e., expiratory flows, PE_MAX_, PI_MAX_) occurred regardless of the starting age of swimming or years of swimming experience. Second, the changes in lung size and function during one competitive swimming season were similar between swimmers and controls. Lastly, despite the swimmers displaying enhanced resting pulmonary size and function, the groups exhibited similar degrees of ventilatory constraint while cycling as evidenced by EELV, frequency and severity of EFL, and utilization of ${\dot{V}}_{\mathrm{ECAP}}$. Collectively, the results of this study suggest that pubertal female swimmers already have greater lung size, expiratory flows, and indices of respiratory muscle strength compared to controls, but similar ventilatory mechanics while cycling. The results support the notion that competitive swim training during periods of peak lung growth does not influence the development of lung size or function, or confer a protective effect from exercise ventilatory constraints.

### Pulmonary function prior to a season of swim training

Compared to controls, swimmers initially had a 20% larger TLC and 25% greater FVC. These differences are congruent with the 20% greater TLC reported by Cordain and Stager in collegiate swimmers (Cordain and Stager 1988) and 20% greater FVC reported by Baxter‐Jones and Helms in pre, peri, and postpubertal swimmers (Baxter‐Jones and Helms 1996). In comparison to reference values, swimmers had an average %‐predicted value ≥110% for initial TLC, FVC, and D_L,CO_ that was not associated with training experience. Interestingly, these initial %‐predicted values were nearly identical to the female members of the 1984 U.S. Olympic swim team for TLC (110 vs. 115%), RV (both 96%), FVC (both 123%), FEV_1_ (117 vs. 116%), and D_L,CO_ (121 vs. 127%) (Bradley et al. 1985). Interpretation of our results in the context of previous investigations of competitive swimmers indicate that the lung size and function of competitive swimmers is already enhanced when they reach puberty, regardless of swimming history. Furthermore, the extent of this enhanced pulmonary profile, relative to either a control group or reference values, is similar whether they are at the beginning of their swimming career during early adolescence or competing at the Olympic level as young adults. Thus, our findings agree with those of others (Baxter‐Jones and Helms 1996) suggesting that enhanced lung size and function are inherent rather than accentuated by competitive swim training.

### Changes in pulmonary function

Over the course of one competitive swim season, we found that anthropometric and absolute pulmonary function measures significantly increased for both swimmers and controls. However, mean growth was not different between swimmers and controls for TLC (0.35 ± 0.13 (SWIM) vs. 0.26 ± 0.25 (CON) L or 8 ± 4 (SWIM) vs. 6 ± 6 (CON) %), spirometry (e.g., FVC: 0.23 ± 0.15 (SWIM) vs. 0.15 ± 0.19 (CON) L; FEF_25‐75%_: 0.21 ± 0.38 (SWIM) vs. 0.10 ± 0.24 (CON) L sec^−1^), and PI_MAX_ and PE_MAX_. Changes in D_L,CO_/*V*_A_ were also similar between groups, decreasing from PRE to POST as expected (Kim et al. 2012). Moreover, %‐predicted values did not change from PRE to POST in swimmers or controls, suggesting that both groups experienced normal lung growth for the given changes in age and body size. Lastly, in swimmers, these changes occurred irrespective of the training volume. Thus, the lack of significant differences in changes in absolute or relative lung size, lung function, or indices of respiratory muscle strength indicate that competitive swimming during puberty did not accentuate lung development.

Analysis of other longitudinal studies supports our finding. Engstrom et al. (1971) reported that TLC was initially larger in female swimmers aged 9–13 years old compared to reference values and did not increase further up to age 16 years old. Baxter‐Jones and Helms found that the FVC of swimmers as young as 8 years old was ≥20% larger than other young athletes at their initial measurement and the difference did not increase further up to 16 years old (Baxter‐Jones and Helms 1996). Furthermore, Andrew et al. (1972) observed a greater TLC, maximal mid‐expiratory flow, and D_L,CO_, but not D_L,CO_/TLC, across all ages in 8‐ to 18‐year‐old swimmers compared to nonathletes. Lastly, Bloomfield et al. (1990) found that FVC was larger in swimmers in a 5 year, mixed‐longitudinal analysis of 8‐ to 12‐year‐old competitive swimmers and nonathletes stratified based on sex and sexual maturity. Congruency between our results and those of four large, longitudinal studies of swimmers throughout all stages of development substantiates our conclusion that the enhanced lung size and function are inherent and not accentuated by competitive swim training.

However, not all studies corroborate our findings. For example, despite not finding a significant interaction between swimming and sexual maturity, Bloomfield et al. (1990) interpreted post hoc tests and stated that FVC became greater in swimmers than nonathletes at pubescent stage 2 in males and stage 4 in females. Vaccaro and Clarke found no difference in FVC between swimmers and controls aged 9–11 years old before or after 7 months of intensive swim training (Vaccaro and Clarke 1978). The reason for our opposing results is unclear given a similar experimental design, but may be related to the controls being 5 cm taller than the swimmers in their study. Longitudinal studies of 9‐10(Courteix et al. 1997) and 7–11 (Zinman and Gaultier 1987) year‐old swimmers concluded that 1 year of swim training caused greater increases in TLC. However, Zinman and Gaultier did not statistically compare their swimmers to a control group (Zinman and Gaultier 1987). In addition, the control group of Courteix et al. (1997) grew 5 cm in height but only 90 mL in TLC, which appears abnormally small for their age and somatic growth. Thus, discrepancies in control groups and experimental analysis, but not effects of competitive swimming, may underlie contrasting conclusions. In the present investigation, swimmers were akin to previous reports of competitive female swimmers for training volume (Zinman and Gaultier 1986; Bloomfield et al. 1990; Baxter‐Jones and Helms 1996; Courteix et al. 1997), swimming history (Zinman and Gaultier 1986), height and weight (Astrand et al. 1963; Wells et al. 2006), and absolute (Astrand et al. 1963; Yost et al. 1981; Zinman and Gaultier 1986; Wells et al. 2006) and %‐predicted (Astrand et al. 1963; Bradley et al. 1985) pulmonary function. The controls also had normal function and lung growth, as evidenced by mean %‐predicted values ranging between 90 and 105% at PRE and POST (Table 1). Therefore, we are confident that the swimmers and controls in this study were representative of their respective populations with regards to lung size and function.

Our study was not designed to address why differences in lung size and function existed; hence, explaining why the swimmers had larger lungs and greater flows is purely speculative. Swimming imposes four unique challenges upon the developing respiratory system; first, it is performed in the prone or supine position with the body partially or fully submerged (Cordain and Stager 1988); second, swimmers use an “obligatory, controlled frequency” breathing pattern (Cordain and Stager 1988); third, training often involves breath control drills, including “hypoxic training”, and sprint swimming where breathing frequency is reduced; and, fourth, intense and structured swim training begins as early as 5 years old (Baxter‐Jones and Helms 1996). While it has been proposed that swimming accentuates lung growth, at present there is no evidence connecting these stressors with the proposed mechanisms of induced postnatal lung growth (for mechanisms, see (Hsia et al. 2004)). Instead, the greater TLC of swimmers may have been related to larger chests. Normal lung development occurs via a feedback loop between rib cage and alveolar growths (Hsia et al. 2004), such that chest wall and lung growths are tightly coupled and their dimensions reach peak growth velocities at similar times (Simon et al. 1972). Moreover, increases in TLC during childhood are due primarily to somatic growth of the chest wall (ATS/NHLBI workshop, 2003). Although not measured in this study, chest wall dimensions were observed to be greater in swimmers as young as 7‐8‐year old (Zinman and Gaultier 1986) that persist throughout adolescence (Bloomfield et al. 1990) and are correlated with a larger TLC (Armour et al. 1993). The larger TLC can also explain why other measures of pulmonary function are greater, including FVC, PEF, mid‐expiratory flows, the MEFV curve, and D_L,CO_. Cumulatively, these may explain why swimmers in this study had enhanced pulmonary function at the initial measurement that did not grow significantly more during one competitive swimming season during puberty.

### Metabolic and ventilatory response to cycle exercise

One season of competitive swimming during puberty did not affect the metabolic responses to exercise. Differences in work rate and $\dot{V}$O_2_ were due to the swimmers being slightly, albeit not significantly, larger in body size. This likely explains the greater $\dot{V}$CO_2_, which led to an increased ${\dot{V}}_{E}$ achieved by a larger *V*_T_. The swimmers had a similar $\dot{V}$O_2MAX_ and maintenance of $\dot{V}$O_2MAX_ after one season of intensive swim training as previously reported 10‐ to 16‐year‐old female competitive swimmers (Robinson et al. 1978). Both groups had excellent exercise capacities as evidenced by a mean $\dot{V}$O_2MAX_ that was 115–131% predicted. The similar peak aerobic capacity is not surprising considering that both groups reported physical activity levels that nearly doubled the Canadian guideline of at least 60 min of daily moderate‐vigorous intensity physical activity (Tremblay et al. 2016).

The swimmers facilitated a larger *V*_T_ by operating at a similar EELV and higher EILV, utilizing a smaller relative portion of their significantly greater IC. However, despite the significantly greater IC in swimmers, ventilatory mechanics were not different between groups. This was because absolute EELV was the same and the MEFV curve was only geometrically larger (quantitative characteristics were not different). Recalling Figure 3, swimmers and controls produced similar flows for a given absolute FVC on the average MEFV curve. Therefore, the groups had similar flow constraints when operating at a similar EELV. This may explain why the swimmers did not operate at a lower relative EELV, they were equally susceptible to EFL, and no significant differences in ${\dot{V}}_{\mathrm{ECAP}}$ and ${\dot{V}}_{E}$/${\dot{V}}_{\mathrm{ECAP}}$ were observed. The qualitative differences in ventilatory constraints can also be seen in the composite MEFV curve and superimposed FVL (Fig. 5). Hypothetically, to alleviate ventilatory constraints by increasing ventilatory capacity or avoiding EFL, the swimmers would have to operate at a higher EELV. However, this would come at the cost of a greater work of breathing due to breathing along a less compliant segment of the pressure–volume curve. The absence of any differences between groups in ${\dot{V}}_{\mathrm{ECAP}}$ also negates the possibility of increased metabolic and ventilatory demands within similar ventilatory constraints. Instead, any increase in demand would necessitate greater susceptibility to ventilatory constraints. Thus, the significantly larger TLC and PEF of competitive swimmers did not affect the occurrence of ventilatory constraints while cycling.

The observation of an increased frequency of EFL from PRE to POST in our subjects differ from those of Emerson et al. (Emerson et al. 2015), who found a decreased frequency of EFL as children mature from pre to postpubescence. They attributed this decline to greater increases in lung volumes and expiratory flows compared to exercise capacity (increasing ventilatory capacity well beyond the metabolic demand) and lowered sensitivity to CO_2_ during exercise (decreasing the ventilatory demand). An explanation for the divergent observations is not obvious, but may relate to differences in study length, subject size, activity level, aerobic fitness, or methods employed for the assessment of EFL. Thus, the question of whether EFL occurs more or less frequently following pubertal growth warrants additional study.

### Limitations

Additional measures, such as partitioning diffusion capacity, assessing chest wall dimensions, or using an esophageal balloon, may have provided mechanistic insight (e.g., lung elastic recoil pressure, compliance, and work of breathing) into physiological differences in the larger lungs of swimmers. Second, because rates of lung growth during development vary substantially depending on age and sexual maturity, it is difficult to ascertain an appropriate sample size for detecting changes in lung growth between swimmers and controls. Given that smaller sample sizes have (Courteix et al. 1997) and larger sample sizes have not (Andrew et al. 1972; Baxter‐Jones and Helms 1996) observed differences in lung growth between swimmers and nonswimmers, it is not clear how a larger sample size would affect our conclusions. Additionally, *P*‐values for many interaction and main effects for operational lung volumes ranged from 0.03 to 0.18; it is possible that a larger sample size or longer time between visits may have affected the statistical significance and therefore interpretation of the operating lung volumes throughout exercise. Lastly, many maneuvers required maximal efforts; given the age of the subjects, submaximal efforts may have underestimated pulmonary function. To minimize poor effort, experienced pediatric Respiratory Therapists performed the pulmonary function test. Nonetheless, effort‐independent techniques, such as negative expiratory pressure (Calverley and Koulouris 2005), may be more suitable to assess ventilatory constraints in children.

## Conclusions

In this study, healthy female competitive swimmers and controls of similar age, sexual maturity, and body size underwent pulmonary size and function and incremental exercise testing before and after one season of competitive swimming. By puberty, competitive swimmers already exhibited enhanced lung size and function compared to controls regardless of the age they started swimming or number of years of experience. One season of training did not further accentuate this enhanced function. Ventilatory responses to cycling exercise were also not different between swimmers and controls; thus, the greater lung size and function of swimmers does not confer a protective effect from exercise ventilatory constraints. Our results support the notions that competitive swimming does not affect lung growth during puberty and the large lungs of swimmers are inherent rather than induced by swimming.

## Conflict of Interest

The authors declare no conflicts of interest. The authors declare that the results of the study are presented clearly, honestly, and without fabrication, falsification, or inappropriate data manipulation.
